# Gelsolin regulates proliferation, apoptosis and invasion in natural killer/T-cell lymphoma cells

**DOI:** 10.1242/bio.027557

**Published:** 2017-11-24

**Authors:** Yanwei Guo, Hongqiao Zhang, Xin Xing, Lijuan Wang, Jian Zhang, Lin Yan, Xiaoke Zheng, Mingzhi Zhang

**Affiliations:** 1 Department of Oncology, The First Affiliated Hospital of Zhengzhou University, Zhengzhou 450052, PR China; 2 Department of Oncology, The Fifth Affiliated Hospital of Zhengzhou University, Zhengzhou 450052, PR China

**Keywords:** Gelsolin, NK/T-cell lymphoma cell, Cell proliferation, Apoptosis, Invasion

## Abstract

The expression of gelsolin (GSN) is abnormal in many cancers, including extranodal nasal-type natural killer/T-cell lymphoma (NKTCL). However, the biological function of GSN and its mechanism in NKTCL remain unclear. We found that GSN overexpression significantly suppressed cell proliferation, colony formation and invasion, and promoted apoptosis of natural killer (NK) cell line YTS. Moreover, the upregulation of GSN significantly decreased the levels of PI3K and p-Akt. Interestingly, blocking the PI3K/Akt signaling pathway significantly inhibited cell proliferation and invasion and promoted apoptosis of YTS cells. In conclusion, our findings indicate that GSN can suppress cell proliferation and invasion and promote apoptosis of YTS cells, and the PI3K/Akt signaling pathway is likely to be involved in this process.

## Abstract (precis)

**Summary:** GSN can suppress cell proliferation and invasion and promote apoptosis of YTS cells, and the PI3K/AKT signaling pathway is likely to be involved in this process.

## INTRODUCTION

Extranodal nasal-type natural killer/T-cell lymphoma (NKTCL) is one of the Epstein-Barr virus (EBV)-related hematological malignancies, which mainly develops in the nasal cavity but can also occur in extranasal sites, either as a primary extranasal or disseminated disease (Harabuchi et al., 1996; Chen et al., 2015). NKTCL is more common in Asia than in Western countries (Au et al., 2009). Although most of the cases of NKTCL are diagnosed in the early stage of the disease, the long-term survival rate of patients is ∼46%-60% (Suzuki et al., 2010). The one-year survival rate of patients with advanced-stage disease is only 50%, despite improvements in treatment (Jaccard et al., 2011; Yamaguchi et al., 2011). The tumor cells of NKTCL derived from NK cells and, rarely, T cells are linked to EBV infection (Huang et al., 2013). However, the biological characteristics of NKTCL are not yet completely clear.

Gelsolin (GSN), a Ca^2+^-regulated actin filament severing and capping protein, is a widespread, polyfunctional regulator of cell structure and metabolism (Li et al., 2012). GSN is a widely expressed actin regulator, and has been reported to be a multifunctional regulator of physiological and pathological cellular processes, and regulates cell migration, cell morphology, proliferation and apoptosis (Sun et al., 1999; Li et al., 2012). Previous research demonstrated that GSN was prevalently expressed in a variety of cells (Tanaka et al., 2006). A previous study revealed that the levels of GSN are decreased in various cancers, including breast, urinary bladder, colon, kidney, ovary, prostate, gastric and urinary system cancer (Tanaka et al., 2006). A study presented by Zhou et al. (2015) showed that upregulated GSN inhibits apoptosis, whereas downregulated GSN promotes apoptosis, which could be associated with the regulation of GSN in the apoptosis-associated pathways and the apoptosis factors caspase 3 and bcl-2. In addition, a study showed that GSN was observed *in vitro* to suppress the proliferation and invasion of 786-0 renal cell carcinoma cells (Zhu et al., 2015). A previous study found that GSN in colorectal tumor cell regulates cell invasion through its modulation of the urokinase (uPA)/urokinase receptor (uPAR) cascade, with possible vital roles in colorectal tumor dissemination to metastatic sites (Zhuo et al., 2012).

GSN displayed high expression in the secondary diffuse large B-cell lymphoma (DLBCL) compared with *de novo* DLBCL (Ludvigsen et al., 2015). However, a recent study revealed that the level of GSN is downregulated in serums of advanced NKTCL patients (Zhou et al., 2016). Although the roles of GSN have been explored, whether the GSN can modulate cell proliferation, apoptosis and invasion in NK/T-cell lymphoma cells is currently unknown. Further investigations are required concerning the role of GSN in NK/T-cell lymphoma progression to determine whether decreased or increased GSN levels in NK/T-cell lymphoma have a direct relationship with tumorigenesis.

It is well known that the PI3K/Akt/mTOR pathway is important and has been successfully targeted in many cancers, including many lymphomas (Westin, 2014). GSN-PI3K-Akt signaling could be involved in regulating the EMT transcription factors (Westin, 2014). GSN has been shown to physically associate with PI3K (Chellaiah et al., 2000) and promote its activity (Singh et al., 1996). An earlier study showed that inhibition of PI3K repressed GSN protein expression and decreased migration and invasion of hepatocarcinoma cells, which suggested that GSN is involved in the PI3K-Akt pathway (Wu et al., 2013).

Here, we investigated the effects of GSN on the proliferation, apoptosis and invasion of NK/T-cell lymphoma cells *in vitro*, and further explored whether GSN exerts its biological function through the PI3K-Akt pathway. Our findings might contribute to the current understanding of the biological functions of GSN in NK/T-cell lymphoma.

## RESULTS

### Overexpression of GSN in transfected YTS cells

**Fig. 1.**
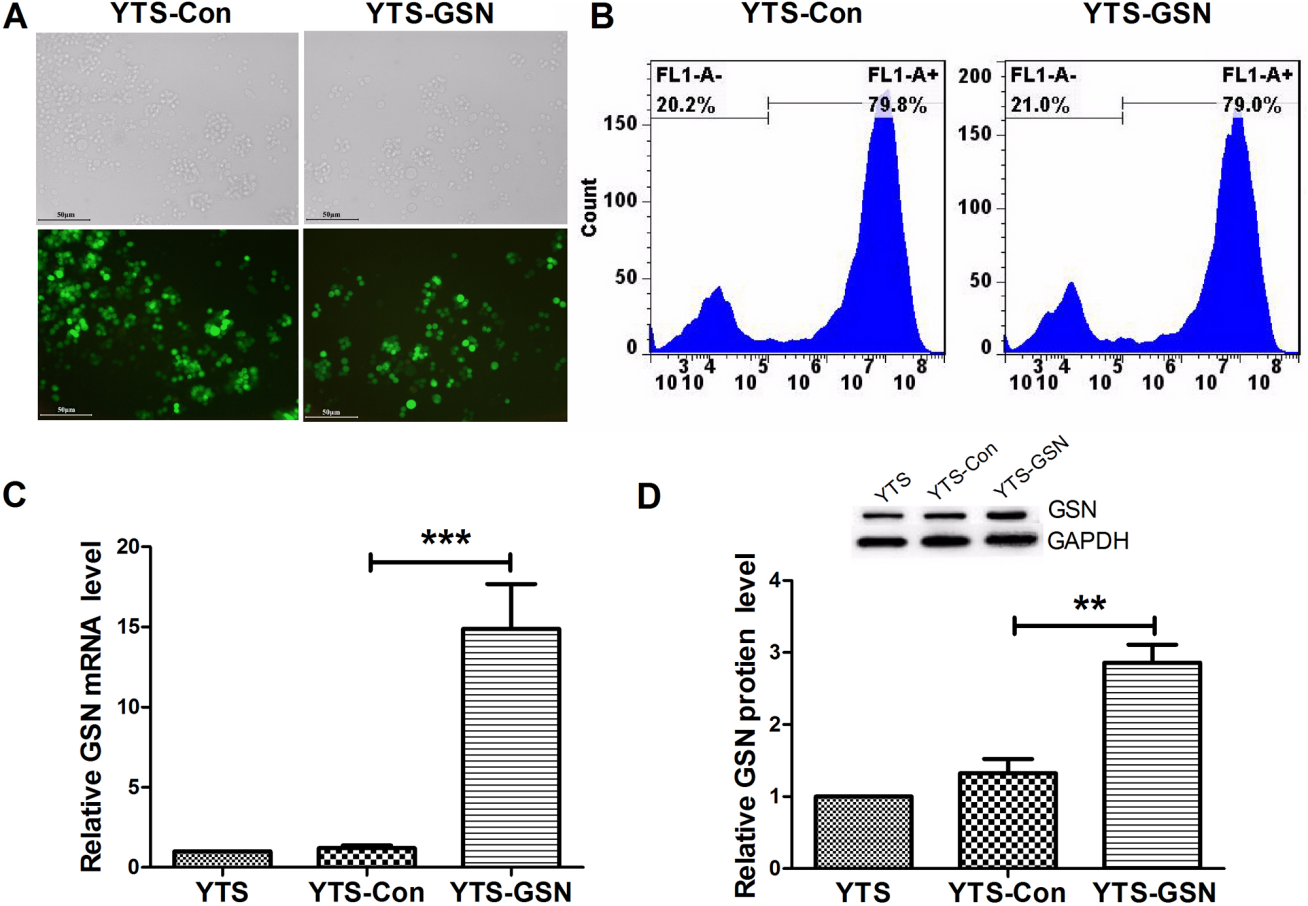
**Transfection ratio of the Lenti-virus-containing Lenti-GSN vector and GSN overexpression in YTS cells.** (A) Green fluorescence was observed in the transfected YTS cells under a fluorescence microscope (×200 magnification) at 48 h, which indicated a successful transfection. (B) Flow cytometric analysis demonstrated that the transfection ratio in cells was 70-80% at 48 h. (C) qRT-PCR analysis exhibited that GSN mRNA expression was higher in YTS-GSN cells than in YTS-Con cells at 48 h. (D) Western blot analysis showed that the level of GSN protein was higher in YTS-GSN cells than in YTS-Con cells at 48 h. GSN, gelsolin; YTS cells, nontransfected cells; YTS-Con cells, YTS cells transfected with the pCDH-CMV-MCS-EF1-copGFP vector; YTS-GSN cells, YTS cells transfected with the pCDH-CMV-MCS-EF1-copGFP-GSN vector. **P<0.01, ***P<0.001.

After the lentivirus-containing Lenti-Con (lentivirus-carrying vectors) and Lenti-GSN (recombinant lentivirus-carrying GSN cDNA) vectors were transfected into natural killer (YTS) cells, green fluorescence was obvious in the infected YTS cells, as observed under a fluorescence microscope, and the result indicated a successful transfection (Fig. 1A). Flow cytometry analysis showed that the transfection ratio in cells was 70-80% (Fig. 1B). Real-time quantitative reverse transcriptase polymerase chain reaction (qRT-PCR) analysis and western blot analysis demonstrated that the mRNA and protein levels of GSN were both significantly increased in the YTS cells transfected with the pCDH-CMV-MCS-EF1-copGFP-GSN vector (YTS-GSN cells), when compared with the YTS cells transfected with the pCDH-CMV-MCS-EF1-copGFP vector (YTS-Con cells) (Fig. 1C,D).

### GSN overexpression inhibits YTS cell proliferation and colony formation

**Fig. 2.**
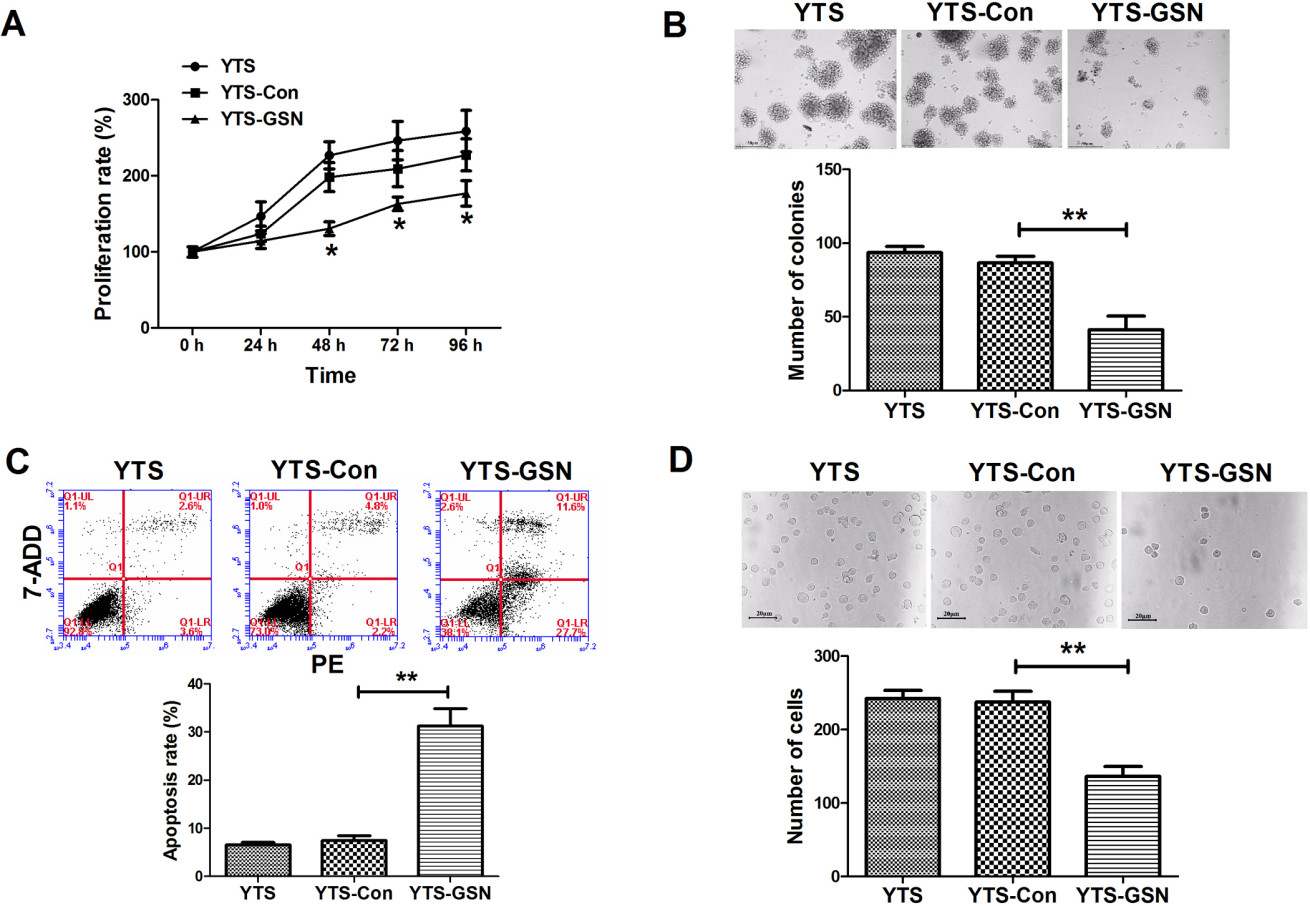
**GSN overexpression inhibits proliferation, colony formation and invasion of YTS cells and promotes apoptosis.** (A) CCK-8 assays revealed that GSN overexpression inhibited YTS cell proliferation. (B) Representative photomicrographs of colony formation assay of YTS cells transfected with lenti-Con and lenti-GSN plasmids for 12 days are shown. Statistical analysis of colony formation assay showed that GSN overexpression caused a decrease in the clonogenic survival of YTS cells compared with YTS-Con cells. (C) Representative photomicrographs of flow cytometric analysis are shown. Statistical analysis of flow cytometric analysis showed that GSN overexpression significantly increased the apoptosis rate in YTS cells at 48 h. The apoptosis rate was the sum of the late apoptosis in the first quadrant and the early apoptosis in the fourth quadrant. (D) Representative photomicrographs of transwell invasion assay in cells at 48 h. Statistical analysis showed that GSN obviously suppressed YTS cell invasion. **P<0.01.

To explore the effects of GSN on YTS cell proliferation and colony formation, CCK-8 and colony formation assays were performed. Our results from CCK-8 assay revealed that cell proliferation of the YTS-GSN cells was significantly suppressed, compared with that of YTS-Con cells (Fig. 2A). In addition, the results of colony formation assay demonstrated that GSN resulted in a decrease in the clonogenic survival of YTS-GSN cells, compared with YTS-Con cells (Fig. 2B). These results suggested that GSN had inhibitory effects on YTS cell proliferation.

### GSN overexpression promotes apoptosis and inhibits YTS cell invasion

Next, we detected the effects of GSN on apoptosis and YTS cell invasion. Flow cytometry analysis revealed that GSN overexpression caused a significant increase in apoptotic YTS cells (Fig. 2C). Transwell assay showed that invasion of YTS-GSN cells was significantly inhibited, compared with YTS-Con cells (Fig. 2D).

### GSN overexpression suppresses the PI3K/Akt pathway in YTS cells

**Fig. 3.**
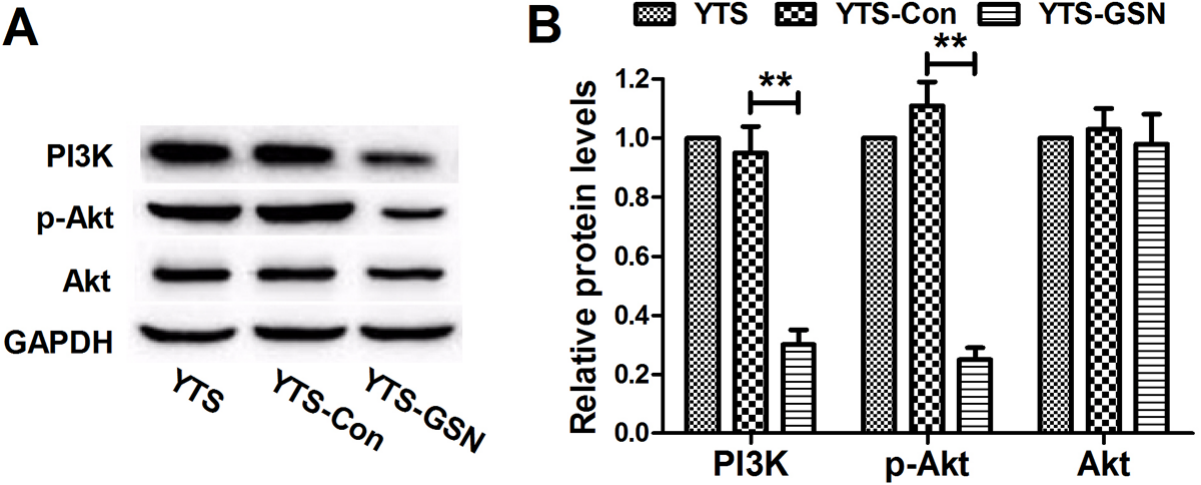
**GSN overexpression inhibits the PI3K/Akt pathway in YTS cells.** (A,B) Western blot analysis revealed that AKT expression in the three experimental groups was not significantly different. The levels of PI3K and p-AKT in YTS-GSN cells were significantly reduced at 48 h, compared with those in YTS-Con cells. **P<0.01.

To further confirm the potential mechanism of the effects of GSN on YTS cells, western blot analysis was performed to detect the components of the PI3K/Akt pathway. As shown in Fig. 3, Akt expression in the three experimental groups was not significantly different. Moreover, phosphorylation of Akt is characteristic of PI3K activation. The levels of PI3K and p-Akt in YTS-GSN cells were both significantly decreased, compared with levels in YTS-Con cells. The results revealed that upregulation of GSN can inhibit the PI3K/Akt pathway.

### Blocking the PI3K/Akt pathway inhibits the proliferation and invasion of YTS cells and promotes apoptosis

**Fig. 4.**
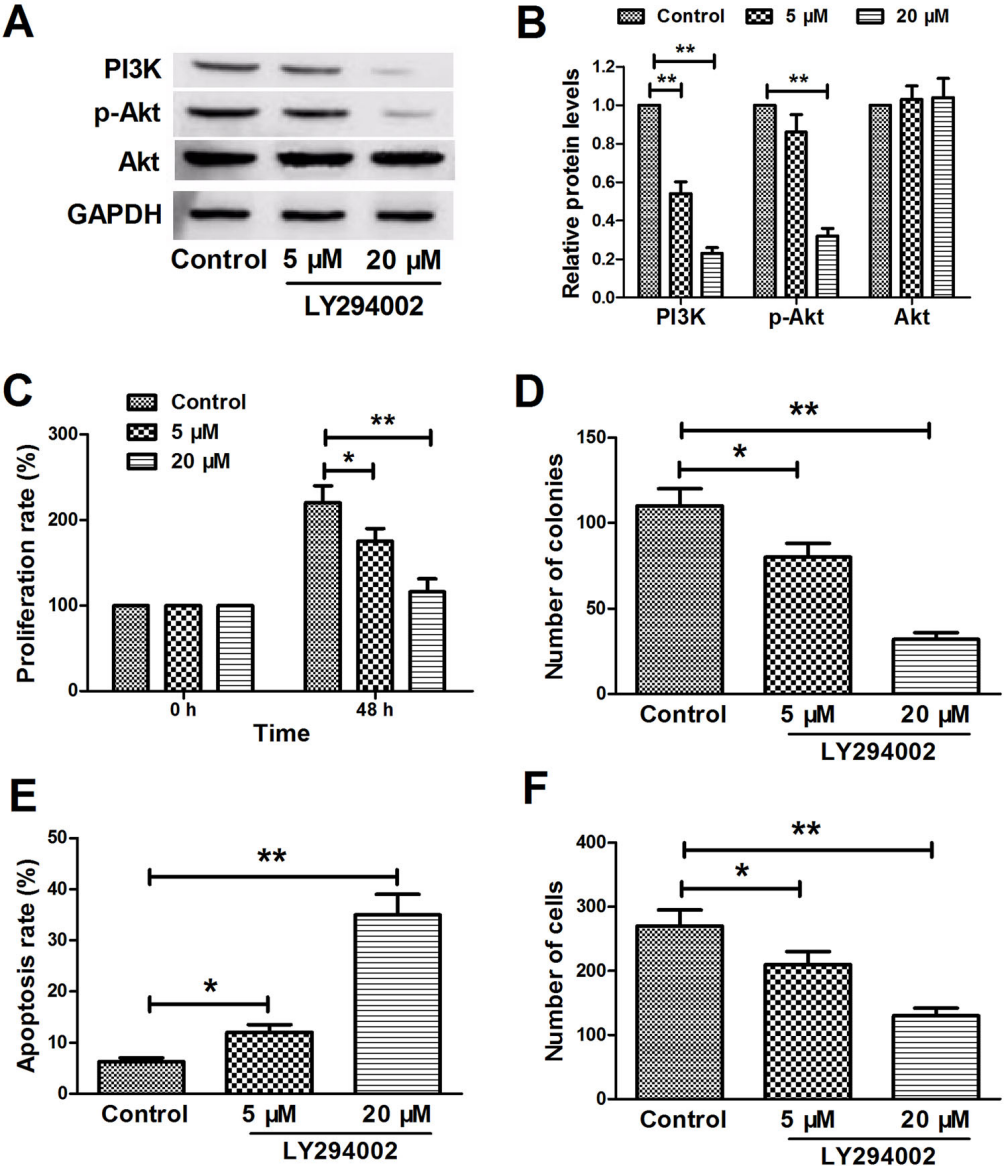
**Blocking the PI3K/AKT pathway inhibits YTS cell proliferation and invasion and promotes apoptosis.** (A,B) Western blot analysis revealed that LY294002 treatment (5 μM and 20 μM) significantly suppressed p-AKT and PI3K in a dose-dependent manner, while AKT expression in the three experimental groups was not significantly different. (C) CCK-8 assay showed that LY294002 treatment had an inhibitory effect on cell proliferation of YTS cells in a dose-dependent manner at 48 h. (D) Colony formation assay showed that LY294002 treatment caused a decrease in the clonogenic survival of YTS cells in a dose-dependent manner. (E) Flow cytometric analysis exhibited that LY294002 treatment had a promotive effect on proliferation of YTS cells in a dose-dependent manner at 48 h. (F) Transwell invasion assay showed that LY294002 treatment had an inhibitory effect on the cell invasion ability of YTS-GSN cells in a dose-dependent manner at 48 h. *P<0.05,**P<0.01.

To confirm whether blocking the PI3K/Akt pathway inhibits cell proliferation and invasiveness and promotes apoptosis of YTS cells, LY-294002, a specific inhibitor of PI3K, which can significantly inhibit the protein expression of p-Akt and PI3K, but not Akt, was used to treat cells (Fig. 4A,B). As expected, CCK-8 and colony formation assays showed that blocking the PI3K-Akt pathway caused increased cell proliferation and colony formation of YTS cells (Fig. 4C,D). Flow cytometry analysis and transwell invasion assay exhibited that blocking the PI3K/Akt pathway resulted in increased apoptosis and diminished cell invasion ability in YTS cells (Fig. 4E,F).

## DISCUSSION

NKTCL is a common kind of malignant lymphoma, and usually develops in the nasal cavity but can also occur in extranasal sites, either as a primary extranasal or disseminated disease (Takeuchi et al., 2014). A recent study demonstrated that the level of GSN is significantly decreased in serums of advanced NKTCL patients (Zhou et al., 2016). However, the potential effects of GSN on NK/T-cell lymphoma cells and molecular mechanisms remain unclear.

GSN is a protein that is broadly expressed intracellularly, including in the cytoplasm and mitochondria, and exists in both intracellular and an extracellular forms (Wen et al., 1996). Previous studies revealed that the expression of GSN is decreased in many cancers, including NKTCL (Tanaka et al., 2006; Zhou et al., 2016). Deng et al. (2015) showed that the upregulation of GSN promotes cell growth and motility and speculate, which is involved in the progression of human oral cancers. Nevertheless, a study has revealed that overexpression of GSN reduces the proliferation and invasion of colon carcinoma cells (Li et al., 2016). Our study results indicate that GSN overexpression significantly suppressed cell proliferation and invasion in YTS cells. A previous study showed that GSN suppresses apoptosis by negatively regulating the expression of apoptosis-associated genes in hepatocarcinoma cells (Zhou et al., 2015). Our results further showed that overexpression of GSN significantly increased apoptosis in YTS cells. Abedini et al. (2014) revealed that GSN plays roles as both an effector and inhibitor of apoptosis, which underlines its association with a wide variety of cancer types. According to the above results, GSN has different effects on cell proliferation, apoptosis and invasion in different cancers, which may be caused by GSN activating or inactivating different signaling pathways in varying cancers.

The PI3K/Akt pathway plays a vital role in cell survival by suppressing apoptosis and promoting cell proliferation (Vivanco and Sawyers, 2002). Akt, an essential serine/threonine kinase, is a crucial component of the PI3K signaling pathway, and its activation has been involved in the genesis or progression of many human malignancies (Blume-Jensen and Hunter, 2001; Vivanco and Sawyers, 2002). Previous studies showed that *AKT1* and *AKT2*, the target genes of PI3K, are overexpressed in breast, gastric and ovarian cancers (Staal, 1987; Bellacosa et al., 1995). Many studies demonstrated that the constitutively active PI3K or Akt is oncogenic in cell systems and animal tumor models (Chang et al., 2003; Liu et al., 2015). Several studies have shown that Akt/PKB is involved in immune activation, cell proliferation, apoptosis and cell survival through activating the transcription of a variety of genes (Fowles et al., 2015; Warfel and Kraft, 2015). Our study revealed that significant upregulation of GSN inhibited the PI3K/Akt pathway in YTS cells. A previous study revealed that the cytoskeletal protein GSN was a vital determinant of cell invasion and scattering by inhibiting E-cadherin expression through the HGF-PI3K-Akt signaling pathway in gastric cancer (Huang et al., 2016). In addition, it has been reported that constitutive PI3K/Akt activation promotes the progress of prostate cancer from an organ-conﬁned disease to a highly invasive and even possibly metastatic disease. Due to its role as a vital regulator of cell survival, Akt has been considered as a crucial factor in tumorigenesis (Nowinski et al., 2015). Consistent with that, in our study, blocking the PI3K/Akt pathway inhibited cell proliferation and invasion of YTS cells, while promoting apoptosis.

## Conclusion

We speculate that GSN overexpression inhibits cell proliferation and invasion and promotes apoptosis of YTS cells, at least partially through suppressing the PI3K/Akt signaling pathway, which is closely related to NKTCL and might have an antitumor effect. However, to our knowledge, relevant reports on the association between GSN and NKTCL are relatively few. Therefore, the specific pathogenesis requires further investigation.

## MATERIALS AND METHODS

### Cell lines and culture

The natural killer (NK) cell line YTS was purchased from American Type Culture Collection (ATCC, Manassas, VA, USA) and maintained in RPMI 1640 medium supplemented with 10% Fetal Bovine Serum (FBS, Takara Biotechnology Co., Ltd., Dalian, China), 1% nonessential amino acids (NEAA, Invitrogen), 1% sodium pyruvate (Sigma-Aldrich), 10 mM HEPES (PAA, Invitrogen), 2 mM L-glutamine (Biochrom, Berlin, Germany), and 1% penicillin-streptomycin (100 μg/ml; Invitrogen Life Technologies, Beijing, China) and 5% CO_2_ at 37°C.

The human embryonic kidney (HEK) 293T cell line was purchased from the cell bank of the Chinese Academy of Sciences (Shanghai, China). The 293T cells were maintained in Dulbecco's Modified Eagle Medium (DMEM, Hyclone, Logan, UT) supplemented with 10% FBS, 10 mM HEPES, 1% penicillin-streptomycin and 5% CO_2_ at 37°C.

### Plasmids

The lentiviral vector used was pCDH-CMV-MCS-EF1-copGFP (DCE; System Biosciences, Mountain View, CA, USA). The packaging plasmids were pCMV-Δ8.2 and pCMV-VSV-G (System Biosciences). The GSN plasmid was purchased from Sino Biological (Beijing, China).

### Construction of the Lenti-GSN vector and lentivirus packaging

A specific primer was designed using Primer Premier 5.0 software (Shanghai Shenggong Biology Engineering Technology Service, Shanghai, China) according to the nucleotide sequences of the human GSN gene, as reported in Genebank (www.ncbi.nlm.nih.gov/genbank/; reference sequence: NM_000177). The primer sequence for GSN was as follows: DCE-GSN-F: 5′-ATTCTAGAGCTAGCGAATTCATGGCTCCGCACCGCCCCG-3′; and DCE-GSN-R: 5′-CCTTCGCGGCCGCGGATCCTCAGGCAGCCAGCTCAGCC-3′. The coding DNA sequence region of the GSN gene was amplified in a thermal cycler (Gene Amp PCR system 2400, Perkin-Elmer, Foster City, CA, USA) according to the manufacturer's instructions. The target DNA gene fragment was subcloned into the DCE lentiviral vector to construct a GSN overexpression lentiviral vector (lenti-GSN).

293T cells were cultured in 10-cm cell culture dishes (3×10^6^ cells/dish). The lentiviral vector packaging system was made as follows: a solution of 500 μl was first prepared consisting of 12 μg plasmid pCMV-Δ8.2, 10 μg pCMV-VSV-G, 22 μg transfer expression plasmid lenti-GSN, and 125 μl 2 mM CaCl_2_ in deionized distilled water. CaCl_2_/DNA was then added dropwise while vortexing to a volume of 2×HEPES-buffered saline (HBS) to a total of 1 ml, and was added to the cells at a density of 80%. GFP expression was observed by fluorescent microscopy after 24 h. The supernatant was harvested by centrifugation at 3000 rpm for 5 min at 4°C after 48 h and the ratio of positive cells was measured by using FACSCalibur flow cytometer (Becton Dickinson, Franklin Lakes, NJ, USA). The high-concentration lentiviral concentrate was used to infect the YTS cells.

### Lentiviral transfection of the YTS cells

YTS cells were seeded in 24-well plates (4×10^4^ cells/well). The viral supernatant with Lenti-Con (lentivirus-carrying vectors) and Lenti-GSN (recombinant lentivirus-carrying GSN cDNA) were added into the cells at a density of 70%-80%, respectively. After 72 h, the transfection ratio was determined under a fluorescence microscope and was measured by flow cytometry. The cells with a transfection ratio of >70% served as the target cells and were identified by qRT-PCR analysis.

### RT-qPCR analysis

Total RNA was isolated from cultured cells using TRIZOL reagent (Invitrogen). 2 μg total RNA was then reverse-transcribed using the Transcriptor First Strand cDNA synthesis Kit (Roche, Mannheim, Germany) with random hexamers. GSN mRNA was detected using Fast SYBR green PCR master mix (PE Applied Biosystems, Foster City, CA, USA) according to the manufacturer's protocol, and the primer sequence for GSN and GAPDH was as follows: GSN-QF: 5′-GCT GAG GTT GCC GCT GGT G-3′, and GSN-QR: 5′-TGT GTT GGT TGC ATT TCC TTT TTG-3′; GAPDH-F: 5′-TGG TAT CGT GGA AGG ACT CAT GAC-3′, and GAPDH-R: 5′-ATG CCA GTG AGC TTC CCG TTC AGC-3′. Relative mRNA expression of GSN was calculated with the comparative threshold cycle (Ct) (2^−ΔΔCt^) method.

### Cell proliferation assay and soft agar colony formation assay

CCK-8 assay was performed to detect the growth of YTS, Lenti-Con-transfected YTS, as well as Lenti-GSN-transfected YTS cells. Cells were seeded in 96-well plates at a density of 1×10^4^ cells/well and incubated for 24, 48, 72 or 96 h in a humidified incubator. Subsequently, 10 μl CCK-8 solution (7Sea PharmTech, Shanghai, China) was added to the wells at the indicated times. After incubation for 3-4 h, absorbance was detected using the using a multilabel counter (Enspire Multimode Plate Reader, PerkinElmer) at 450 nm.

For colony formation assays, all six-well culture plates containing the bottom and soft layers were used. The cells were plated in soft agarose as follows: cells were harvested from monolayer culture, washed and resuspended at 4×10^4^ cells/ml in fully supplemented RPMI 1640 culture medium and molten 1.5% agarose (to a final concentration of 0.3%) on Day 0, then 0.5 ml of the cellular suspension was applied to the base layer (1×10^4^ cells/well) and allowed to set at 4°C for 6 min. Duplicate soft agarose cultures were established to assess colony formation. Cultures were placed in an incubator at 37°C, 5% CO_2_ and 100% relative humidity for 12 days. The number of colonies containing ≥50 cells was counted using a light microscope.

### Cell apoptosis assay

Cell apoptosis was detected using the Annexin V-phycoerythrin (PE)/7-amino-actinomycin D (7-AAD) Apoptosis Detection Kit (Nanjing KeyGen Biotech, Nanjing, China) according to the manufacturer's instructions. Cells were seed in six-well plates at 5×10^5^ per well. The cells were harvested and washed twice in PBS. Then, 1×10^6^ cells were resuspended in 500 μl binding buffer. The suspension was stained with 1 μl Annexin V-PE in the dark for 10 min at room temperature, and 5 μl 7-AAD was added to the suspension and maintained for 10 min at room temperature in the dark. Cell apoptosis was analyzed using a BD FACSAria II cell sorter (BD Biosciences, Franklin Lakes, NJ, USA).

### Transwell invasion assay

For invasion assays, transwell filters (Corning Incorporated, Corning, NY, USA) were coated with Matrigel (BD Biosciences) for 24 h, and 2×10^5^ cells were seeded into the upper compartment of the chambers with 100 μl serum-free RPMI-1640 medium. The lower chamber of the transwell was filled with culture media containing 10% FBS as a chemo-attractant. After 48 h incubation, noninvaded cells on the top of the transwell were scraped off with a cotton swab. Cells successfully translocated were fixed with 10% formalin and counted under a light microscope.

### Western blot analysis

Total protein was extracted from cells using lysis buffer (Roche Diagnostics, Basel, Switzerland). Protein samples (30 μg) were separated by 10% SDS-PAGE and transferred onto polyvinylidene fluoride (PVDF) ultrafiltration membrane (Sigma-Aldrich) for 2 h at 4°C. The membranes were blocked with 5% nonfat milk for 1 h at room temperature. The membranes were washed three times for 5 min each with 15 ml TBS Tween 20 (TBST; Cell Signaling Technology). The membranes were incubated with primary antibodies overnight at 4°C. The membranes were then incubated with horseradish peroxidase (HRP)-conjugated antibody for 2 h at 37°C. Antigen-antibody complexes were visualized by enhanced chemiluminescence (ECL) blotting analysis system (Amersham Pharmacia Biotech, Buckinghamshire, UK) and GAPDH served as the internal reference. The primary antibodies used in this study are as follows: GSN, PI3K, Akt, p-Akt and GAPDH antibody (1:1000; Cell Signaling Technology). An HRP-conjugated anti-rabbit IgG antibody was used as the secondary antibody (Santa Cruz Biotechnology).

### Statistical analysis

Data are presented as the mean±s.d. and were analyzed using *t*-test or analysis of variance (ANOVA) by SPSS Software version 19 (SPSS, Chicago, IL, USA). *P*<0.05 was considered to be statistically significant.
